# Fatty acid composition of adipose tissue at term indicates deficiency of arachidonic and docosahexaenoic acid and excessive linoleic acid supply in preterm infants

**DOI:** 10.1007/s00394-020-02293-2

**Published:** 2020-05-31

**Authors:** K. A. Böckmann, A. von Stumpff, W. Bernhard, A. Shunova, M. Minarski, B. Frische, S. Warmann, E. Schleicher, C. F. Poets, A. R. Franz

**Affiliations:** 1 Department of Neonatology, Faculty of Medicine, Eberhard-Karls-University, Calwer Straße 7, 72076 Tübingen, Germany; 2 Center for Pediatric Clinical Studies, Faculty of Medicine, Eberhard-Karls-University, Tübingen, Germany; 3 Department of Pediatric Surgery and Child Urology, Faculty of Medicine, Eberhard-Karls-University, Tübingen, Germany; 4 Department of Internal Medicine IV, Faculty of Medicine, Eberhard-Karls-University, Tübingen, Germany

**Keywords:** Adipose tissue, Arachidonic acid, Docosahexaenoic acid, Polyunsaturated fatty acids, Preterm infants, Triglycerides

## Abstract

**Background:**

Arachidonic (ARA) and docosahexaenoic acid (DHA) are constitutive to membrane phospholipids, and essential for brain and overall development. ARA/DHA pools in term infants (TI) are built during the third trimester, stored as adipose tissue triglycerides and predominantly distributed via plasma phosphatidylcholine (PC). In preterm infants (PTI), placental ARA/DHA supply is replaced by linoleic-acid (LA)-enriched nutrition. This study aimed to investigate the impact of PTI nutrition, compared to placental supply, on fatty acid composition in adipose tissue and blood.

**Methods:**

Prospective observational study (4/2017–3/2019) in 12 PTI and 3 PTI with enterostomy (PTI/E) (gestational age (GA) < 32 weeks) with surgical intervention at term (± 6 weeks) and 14 TI (GA ≥ 34 weeks, surgical intervention < 2 weeks postnatally). PTI/E were analyzed descriptively only. PC and triglyceride fatty acids were analyzed with tandem mass spectrometry and gas chromatography, respectively. Results were compared between TI and PTI with Wilcoxon Test and shown as median [25th percentile–75th percentile] mol%.

**Results:**

PTI had less ARA in adipose tissue TG (0.77[0.67–0.87]% vs. 1.04[0.95–1.14]%, *p *= 0.0003) and plasma PC (20.7[18.7–22.8]% vs. 28.3[22.7–33.5]%, *p *= 0.011) than TI. PTI also had less DHA in adipose tissue TG (0.6[0.4–0.8]% vs. 1.1[0.8–1.4]%, *p *= 0.006) and plasma PC (6.4[5.6–7.1]% vs. 8.4[7.8–13.1]%, *p *= 0.002). LA was increased in PTI’s adipose tissue TG (10.0[8.8–12.3]% vs. 3.0[2.5–3.6]%, *p *< 0.0001) and plasma PC (48.4[44.6–49.6]% vs. 30.6[24.9–35.6]%, *p *= 0.0002). Similar differences were observed in erythrocyte PC.

**Conclusion:**

In PTI, LA is increased and ARA/DHA decreased in adipose tissue, plasma and erythrocyte lipids as proxies for other tissues, likely caused by PTI nutrition. This may contribute to impaired PTI development.

**Electronic supplementary material:**

The online version of this article (10.1007/s00394-020-02293-2) contains supplementary material, which is available to authorized users.

## Introduction

Long-chain polyunsaturated fatty acids (LC-PUFA) are important components of glycerophospholipids like phosphatidylcholine (PC) and phosphatidylethanolamine (PE), the main phospholipids in plasma membranes and lipoproteins [1]. Docosahexaenoic (DHA = C22:6 N–3) and arachidonic acid (ARA = C20:4 N–6) are the major LC-PUFA, and are essential for overall organ and particularly cerebral development and homeostasis. Consequently, their alterations in preterm infants are associated with neonatal morbidity [2]. During cerebral growth, both ARA and DHA are enriched in brain tissue. Membrane fluidity, neuro- and synaptogenesis are triggered by DHA, which is the major omega-3 fatty acid in vertebrates [3]. Moreover, DHA and its derivatives, like elovanoids, improve the activity of retinal receptors in a concerted action with retinoid receptors and G-proteins, and reduce oxidative stress and inflammatory processes [4–10]. Because synthesis during rapid fetal and neonatal growth is not sufficient for developmental requirements, these fatty acids are largely supplied during the third trimester via the placenta, and more than 90% of DHA and ARA accretion is deposited in adipose tissue triglycerides (TG) [11].

ARA, as an omega-6 LC-PUFA, is present in cell membrane phospholipids of all organs, and also in the phospholipid fraction of plasma lipoproteins. It is important for growth, cell signal systems, and as a precursor of eicosanoids [12–15]. Notably, in most tissues, ARA concentrations are much higher than those of DHA [16–21]. In the central nervous system, a significant part of ARA is elongated to docosatetraenoic acid (DTA = C22:4n–6), so that in brain gray matter, the sum of ARA and DTA (17.5% of all fatty acids) surmounts DHA values (8.5%). In human neonatal retina, ARA plus DTA equal DHA values (13.3% and 12.3%, respectively) [22].

During the third trimester, total body fat mass grows exponentially, reaching its maximum just before term birth [23]. Comparative analyses of cord and maternal blood reflect the placental transport of ARA and DHA to the fetus, with ARA being increased over maternal values throughout pregnancy (24-week GA onwards) and DHA increasing beyond 32-week GA. By contrast, LA is lower in fetal than in maternal plasma, indicating that LA is retained in the maternal circulation [2, 23, 24]. However, preterm infants (PTI), especially extremely low gestational age newborns (ELGANS), are born with very little adipose tissue and cannot benefit from selective placental DHA and ARA accretion. Instead, placental nutrition is replaced by parenteral or enteral feeding rich in LA, with low or absent DHA and ARA. Consequently, compared to cord plasma, LA-PC increases, whereas DHA- and ARA-PC decrease by 50% in PTI plasma during the first week after birth [24, 25].

Against this background, we aimed to verify the hypothesis that adipose tissue of PTI at term-equivalent age is poorer in DHA and ARA and richer in LA than adipose tissue of TI. Due to the high turnover of lipid metabolism [26, 27], we anticipated that fatty acid composition in plasma and erythrocytes might reflect that of adipose tissue. If representative of other organs, this would indicate a major disturbance of these infants’ whole lipidome.

## Methods

This is a prospective observational study in 15 PTI and 14 term or near-term infants (TI) who had a clinically indicated surgical intervention at term-equivalent age (± 6 weeks). The study was carried out in the neonatal and pediatric surgical departments of the University Hospital Tübingen, Germany. Patients were recruited from April 2017 to March 2019. The Institutional Review Board (project number 006/2017BO1) approved the protocol and written informed parental consent was obtained prior to enrolment.

### Inclusion criteria

Three pre-defined groups of infants were studied: *(Near*-*)TI* with gestational age (GA) at birth ≥ 34 ± 0/7 weeks, with clinically indicated surgical intervention soon after birth (postnatal age (PNA) at surgery < 14 days). This GA range was required because most infants with abdominal wall defects or hydrocephalus are delivered before term. The postnatal age at surgery was chosen because surgical intervention is predominantly, yet not always, in the first week of life (Table 1). *PTI* with GA at birth of 23+0/7 to 31+6/7 weeks, without gastrointestinal problems, and with clinically indicated surgery (e.g., herniotomy) at term-equivalent age (± 6 weeks, i.e., at a postmenstrual age of ≥ 34 ± 0/7 weeks, and a postnatal age of > 14 days). *Preterm infants with enterostomy (PTI/E)* with GA at birth 23+0/7 to 31+6/7 weeks with enterostomy because of necrotizing enterocolitis and/or intestinal perforation and enterostomy closure at term-equivalent age ± 6 weeks. As defined a priori, these PTI/Es were considered separately because we anticipated prolonged periods of parenteral fat supply from SMOFlipid^®^ (Fresenius Kabi, Bad Homburg, Germany) and Omegaven^®^ (Fresenius Kabi) as well as potential malabsorption/intestinal losses of related nutrients through the enterostomy before adipose tissue sampling, potentially confounding lipid homeostasis. While it was initially intended also to collect adipose tissue and blood samples in the PTI/E group during the initial laparotomy for necrotizing enterocolitis and/or intestinal perforation, this turned out to be impractical, because the team felt that asking for study consent was inappropriate in the respective emergency situation. Consequently, all three groups were sampled only once at term-equivalent age (± 6 weeks).

**Table 1:** Demographic data and indication for surgery

	Term infants	Preterm infants	Preterm infants with enterostomy
Number	14	12	3
Gender (m/f)	8/6	11/1	2/1
Birth weight (g)	3015 [2740–3432.5]	907.5 [732.5–1118.8]	730 [485–920]
GA at birth (weeks)	38.2 [36.7–39.3]	26.6 [25.4–27.9]	27.3 [25.6–28.3]
PNA at surgery (weeks)	0.14 [0–0.68]	10 [9.07–11.1]	16 [15.9–18.9]
GA at surgery (weeks)	38.3 [36.7–39.6]	36.6 [35.4–38.4]	44.1 [41.6–46.1]
Indication for surgery			
Inguinal hernia repair	0	11	2
Enterostomy closure	0	0	2
Hydrocele repair	0	1	0
Omphalocele	4	0	0
Gastroschisis	2	0	0
Esophageal atresia	3	0	0
Diaphragmatic hernia	1	0	0
Duodenal atresia	2	0	0
Spina bifida	1	0	0
Congenital pulmonary airway malformation	1	0	0

Data are shown as median [25th percentile–75th percentile], *m* male, *f* female, *GA* gestational age, *PNA* postnatal age, term infants: GA ≥ 34 ± 0/7 weeks, preterm infants and preterm infants with enterostomy: GA 23+0/7 − 31+6/7

*Exclusion criteria* were metabolic and genetic disorders and missing parental consent.

*Postnatal nutrition* occurred according to local standards as previously described [28]. Infants were fed expressed breast milk and/or formula starting at day one. Enteral feeding was increased by 20–30 ml/kg/d, as tolerated, and breast milk was supplemented with commercial fortifiers to meet recommended intakes [29]. Consequently, partial parenteral nutrition (Amino acids, glucose, electrolytes and fat emulsion) starting at day 1 and complementing enteral nutrition until full feeds were reached was usually discontinued at days 5–7 (only the PTI/E infants received prolonged parenteral nutrition).

### Adipose tissue and plasma sampling

During surgery, 1–3 small pieces (2 × 2×2 mm) of subcutaneous adipose tissue were collected. These were transferred into sample containers, submersed in liquid nitrogen to remove oxygen completely, closed and then kept at −80 °C until analysis. During clinically indicated peri-surgical blood sampling, 200 µl of EDTA-blood was collected, immediately centrifuged at 1000 × *g* at room temperature for 10 min, plasma and erythrocyte pellet separated and both stored at −80 °C until analysis.

Outcomes were the molecular compositions of fatty acids in TG and PC in mol% in adipose tissue (primary outcome) and in plasma and erythrocytes (secondary outcomes).

### Materials

Chloroform (HPLC grade) was from Baker (Deventer, The Netherlands). Methanol and water (analytical grade) were from Fluka Analytical/Sigma-Aldrich (Munich, Germany). Internal phospholipid standards, 1,2-diarachidoyl-sn-glycero-3-phosphocholine (diarachidoyl-phosphatidylcholine, PC20:0/20:0) and 1,2-dimyristoyl-sn-glycero-3-phosphoethanolamine (dimyristoyl-phosphatidylethanolamine, PE14:0/14:0) were purchased from Avanti Polar Lipids (Alabaster, Alabama, USA). Purity of chemicals was checked by liquid chromatography heated electrospray ionization tandem mass spectrometry (LC–H-ESI-MS/MS) (see below). All further chemicals were of analytical grade and from various commercial sources.

### Lipid analysis

Adipose tissue, plasma and erythrocyte membranes were extracted with chloroform:methanol according to Bligh and Dyer as previously described [24, 30, 31]. Erythrocyte membranes were prepared by suspending 100-µL erythrocyte pellet in 5-mL double-distilled, degassed water (4 °C). Samples were homogenized, kept for 15 min at 4 °C, and then centrifuged at 3000 × *g* for 20 min. The supernatant was decanted and the pellet used for extraction.

Phospholipid molecular species were analyzed by LC–H-ESI-MS/MS, using PC20:0/20:0 and PE14:0/14:0 as internal standards. The equipment comprised a Finnigan Surveyor Autosampler plus MS Pump plus TSQ Quantum Discovery MAX equipped with heated electrospray ionization interface (H-ESI)) (Thermo Fisher Scientific, Dreieich, Germany), as previously described [32]. PC, lyso-PC and SPH were separated from other phospholipids with a Polaris 3 Si-A column (2.0 × 100 mm; Agilent Technologies, Böblingen, Germany) at 40 °C, using a mobile phase consisting of chloroform:methanol:300-mM ammonium acetate (60:38:2, vol/vol) at flow rates of 400 µL/min (0–0.05 min) → 600 µL/min(0.06–1.39 min) and 400 µL/min (1.40–7.00 min). Individual molecular species were analyzed at positive ionization in the selected reaction monitoring (SRM) mode, using phosphorylcholine (mass/charge [m/z] = + 184) as the diagnostic fragment for individual PC molecular species. Concentrations were calculated relative to their internal standards, and data corrected for the ^13^C effect of components differing in number of carbon units, and for differences in ionization rate according to chain length as described before [24]. PC molecular species were grouped together (see online supplement, Table e1), depending on the presence of two saturated fatty acids (sat-PC), or a mono-unsaturated oleic (OA/C18:1-PC/PE), di-unsaturated linoleic (LA/C18:2-PC), arachidonic (ARA/C20:4-PC) or docosahexaenoic acid (DHA/C22:6-PC) residue, which accounted for > 95% of whole PC.

Reproducibility of analyses was assessed by external standard (control plasma), and 95% confidence intervals for PC species and sub-group composition and concentration are provided in the online supplement (Table e1).

### Triglyceride and fatty acid analysis

Neutral lipids were separated from phospholipids using the chloroform phase of Bligh & Dyer extracts and disposable 100-mg NH2-propyl cartridges (Strata^®^, Phenomenex, Aschaffenburg, Germany) as described before. Fatty acids were determined as fatty acid methyl esters (FAME) after transmethylation using docosatrienoic acid (C22:3n−3) as previously described [33], and 1 µL injected into a HP5890 gas chromatograph (GC) (Hewlett Packard) equipped with a flame ionization detector (250 °C), hydrogen/synthetic air as combustion gases, and a 100 m × 0.25 mm × 0.20 µm HP-88 column (Agilent, Germany) with helium as carrier gas. Column settings were 130 °C for 3 min, and then linearly increased to 176 °C at 27 min, 186 °C at 37 min, 190 °C at 58 min and 220 °C at 92 min. This allowed for the separation of the fatty acids indicated in the results. Quantification was performed using calibration curves for the respective fatty acid methyl esters. Reproducibility of fatty acid quantification is provided in the online supplement (Table e2).

### Statistics

Sample size calculation was done assuming that the proportions of ARA and DHA in adipose tissue PC reflect the previously reported postnatal changes in plasma PC in preterm infants [24], but with slightly lower values (i.e. ~ 90%). Hence, we anticipated a proportion of DHA in adipose tissue TG in TI of 10 ± 2%, in PTI of 5 ± 2%, ARA-TG in TI of 30 ± 5% and in PTI of 20 ± 5%. The primary hypothesis was (DHA/ARA in TI) ≠ (DHA/ARA in PTI = DHA/ARA in PTI/E). Assuming normal distribution of the data, seven patients were required per group for an analysis of variance with post hoc test and partially connected groups to test the primary hypothesis for DHA and ARA (power = 90%, alpha = 2.5%). The level of significance alpha 2.5% results in a global level of significance of 5% when two tests are done (one for DHA and one for ARA) in the same cohort.

Because only 3 patients were recruited into the PTI/E group, PTI/E infants were analyzed descriptively only. Because proportions of DHA and ARA were not normally distributed (according to Shapiro–Wilk test), between-group comparisons (restricted to TI versus PTI) were performed by Wilcoxon test. Data are shown as median [25th percentile–75th percentile]. JMP (Version 13.0.0, SAS Institute GmbH, Germany) was used for statistical analysis. The correlation of fatty acid concentrations and postnatal age was analyzed visually. Graphs and tables were created with Excel 2007, Word 2007 (Microsoft Corporation, USA) and JMP 13.0.0.

## Results

Between April 2017 and March 2019, 120 infants, cared for in the Neonatal Department of University Children’s Hospital Tübingen, had at least one surgical intervention; 33 infants met exclusion criteria, in 10, parents refused to participate in the study, 43 patients were missed (because the study team did not know about the operation or could not pick up the tissue samples), and 5 were recruited but no samples collected (organizational issues (*n *= 3) or tissue samples contained no fat (*n *= 2)). Demographic data of the 29 study patients and indications for surgery are shown in Table 1.

Until the day of surgery, PTI/E infants (*n *= 3) had received SMOF^®^ (Fresenius Kabi) lipid for a median duration of 28 days (cumulative fat intake from SMOF-lipid in g/kg: 35[33.3–54.4]) and Omegaven^®^ (Fresenius Kabi) for 21 days (cumulative fat intake from Omegaven: 18.7[14.2–35.9] g/kg). The PTI group only received SMOF-lipid for 4.5 days after birth (cumulative fat intake from SMOF-lipid 5.6[3.7–8.4] g/kg) and no Omegaven at all.

### Fatty acid composition in adipose tissue and blood

**Fig. 1:**
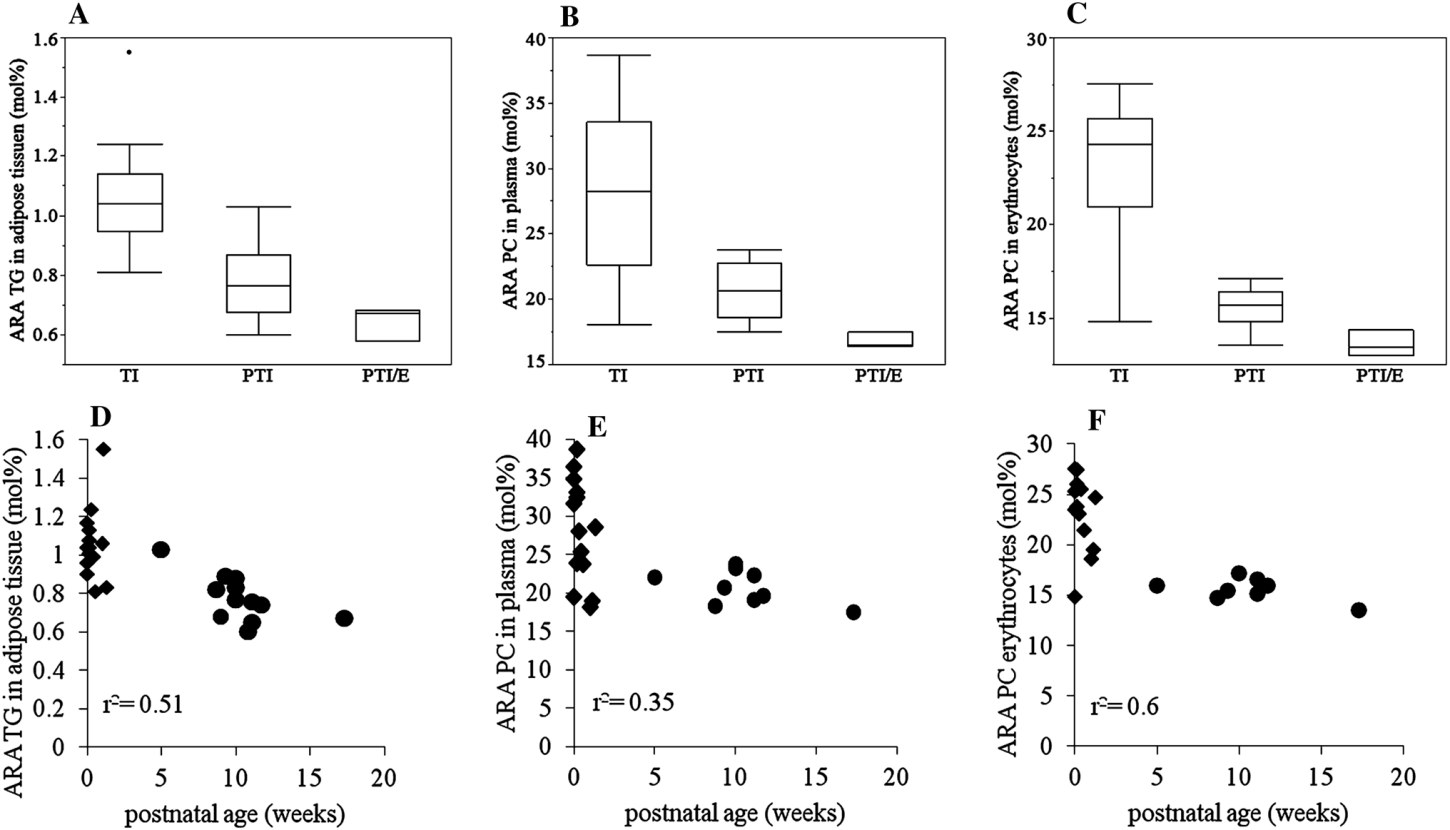
Arachidonic Acid (ARA) in adipose tissue, plasma and erythrocytes. Panels **a**–**c** show the proportion of ARA in mol% in adipose tissue triglycerides (TG), plasma phosphatidylcholine (PC) and erythrocyte PC, respectively, each in term infants (TI), preterm infants without gastrointestinal problems (PTI), and preterm infants with enterostomy (PTI/E). Panels **d**–**f** show the proportion of ARA in mol% to postnatal age in adipose tissue TG, plasma PC and erythrocytes PC in PTI and TI (PTI/E are not shown). All blood and adipose tissue samples were collected during clinically indicated surgery at term-equivalent age. filled diamond = term infants, filled circle = preterm infants

PTI, compared to TI, had less ARA in TG of adipose tissue (*p *= 0.0003). This difference also applied to adipose tissue PC (*p *= 0.03). Similarly, the proportions of ARA-PC in plasma (*p *= 0.011) and erythrocyte membranes (*p *= 0.0007) were lower in PTI than in TI (Fig. 1, panels a–c). The fraction of ARA in plasma TG, however, did not differ between groups (*p *= 0.073). The proportion of ARA decreased with postnatal age in adipose tissue TG, plasma PC and erythrocyte PC (Fig. 1, panels d–f).

**Fig. 2:**
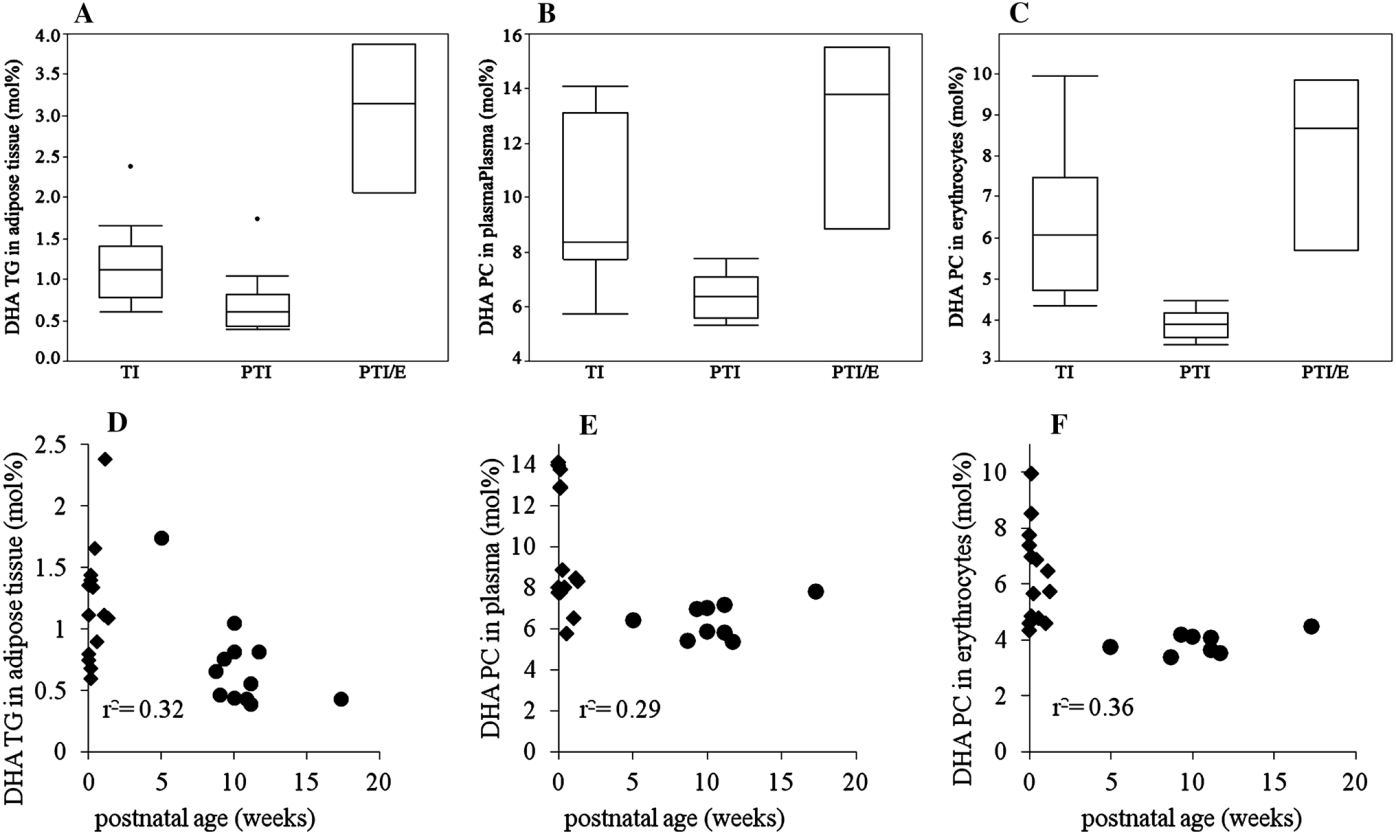
Docosahexaenoic Acid (DHA) in adipose tissue, plasma and erythrocytes. Panels **a**–**c** show the proportion of DHA in mol% in adipose tissue triglycerides (TG), plasma phosphatidylcholine (PC) and erythrocyte PC, respectively, each in term infants (TI), preterm infants without gastrointestinal problems (PTI), and preterm infants with enterostomy (PTI/E). Panels **d**–**f** show the proportion of DHA in mol% to postnatal age in adipose tissue TG, plasma PC and erythrocytes PC in PTI and TI (PTI/E are not shown). All blood and adipose tissue samples were collected during clinically indicated surgery at term-equivalent age. filled diamond = term infants, filled circle = preterm infants

Similar differences were observed for DHA. Here, its proportion in adipose tissue TG of PTI was lower than that of TI (*p *= 0.006). Similarly, in PTI compared to TI, DHA-PC of adipose tissue (*p *= 0.0005), plasma (*p *= 0.002) and erythrocyte membranes (*p *= 0.0002) and the DHA fraction of plasma TG (*p *= 0.001) were lower (Fig. 2, panels a–c). As for ARA, the proportion of DHA in adipose tissue TG, and that of DHA-PC in plasma and erythrocytes decreased with postnatal age (Fig. 2, panels d–f).

**Fig. 3:**
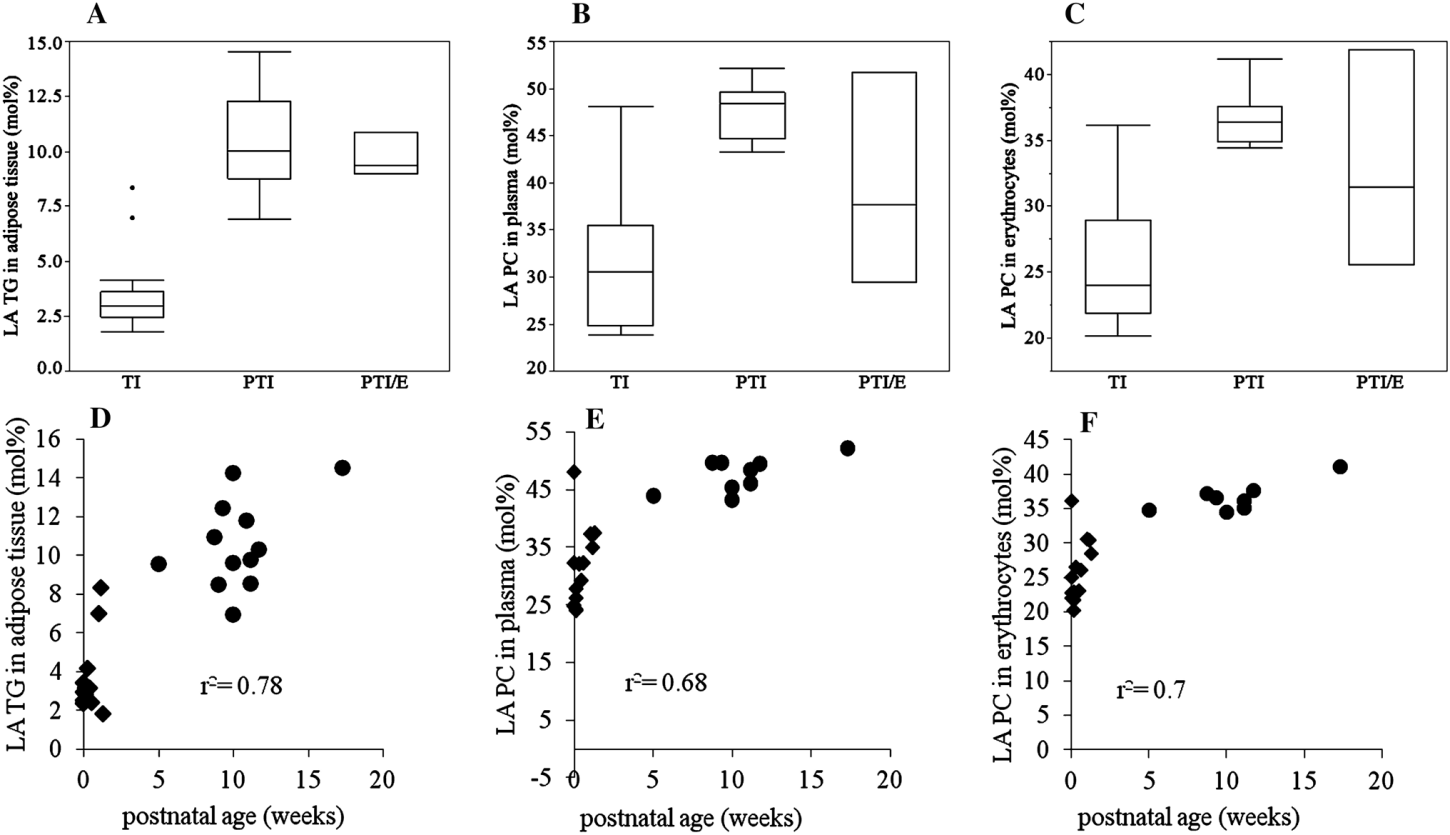
Linoleic Acid (LA) in adipose tissue, plasma and erythrocytes. Panels **a**–**c** show the proportion of LA in mol% in adipose tissue triglycerides (TG), plasma phosphatidylcholine (PC) and erythrocyte PC, respectively, each in term infants (TI), preterm infants without gastrointestinal problems (PTI), and preterm infants with enterostomy (PTI/E). Panels **d**–**f** show the proportion of LA in mol% to postnatal age in adipose tissue TG, plasma PC and erythrocytes PC in PTI and TI (PTI/E are not shown). All blood and adipose tissue samples were collected during clinically indicated surgery at term-equivalent age. filled diamond = term infants, filled circle = preterm infants

By contrast, in lipids of PTI, the proportion of LA was generally higher than in TI. This applied to TG (*p *< 0.0001) and PC (*p *= 0.0002) of adipose tissue as well as to PC of plasma (*p *= 0.0002) and erythrocyte membranes (*p *= 0.0003). LA in plasma TG was not different between groups (*p *= 0.083) (Fig. 3, panels a–c). The proportion of LA increased with postnatal age in adipose tissue TG and in PC of plasma and erythrocytes (Fig. 3, panels d–f).

The result of the PTI/E group (*n *= 3) is shown in Figs. 1, 2, 3a–c) (and in the online supplement Fig. e1–6), with higher fractions of DHA in adipose tissue TG as well as PC of plasma and erythrocyte membranes than in PTI and TI. However, proportions of ARA were decreased and those of LA similar to PTI values.

**Fig. 4:**
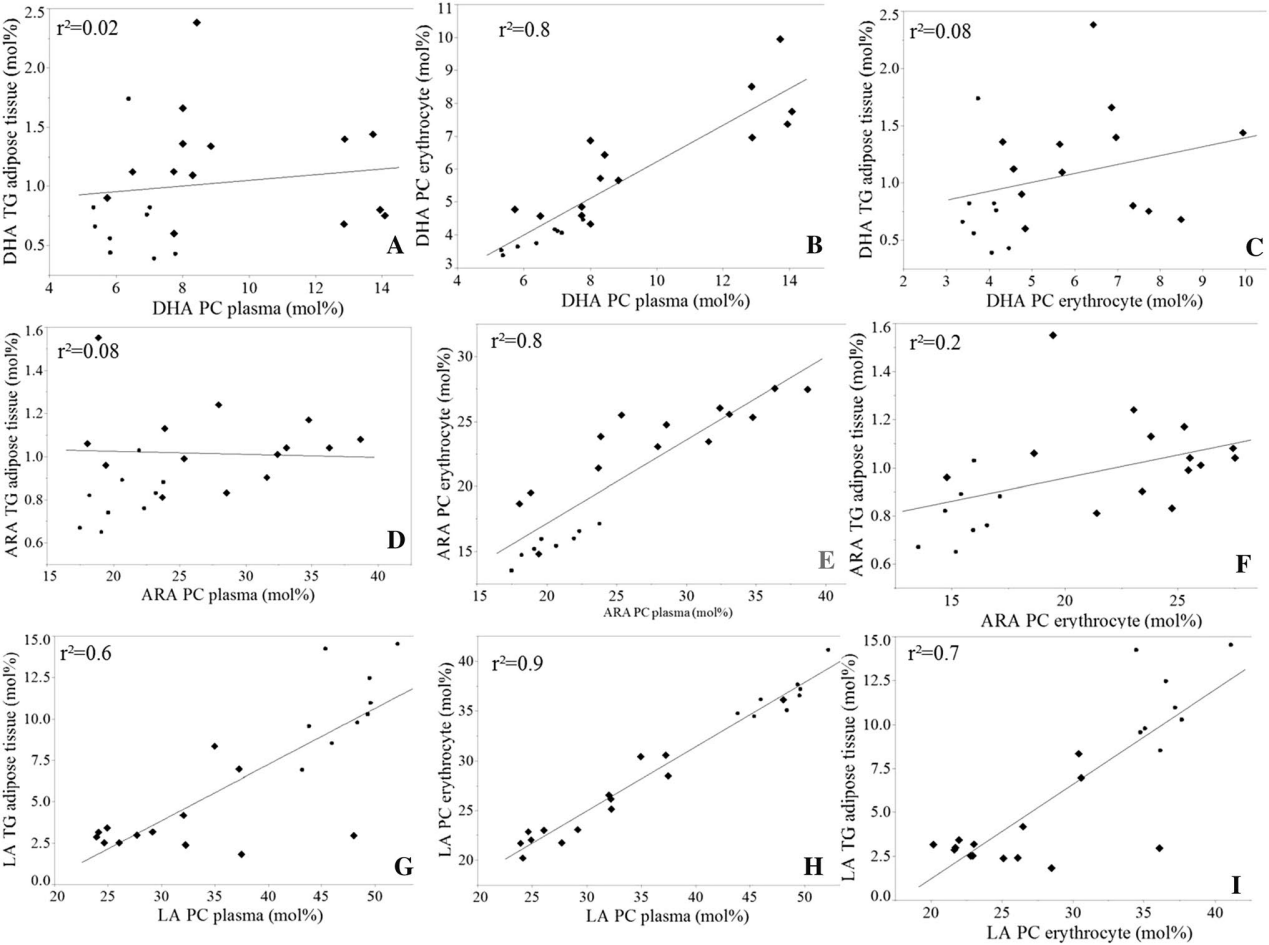
Docosahexaenoic acid (DHA), arachidonic acid (ARA) and linoleic acid (LA) correlations of the different compartments: adipose tissue, plasma and erythrocytes. Panels **a**–**i** shows the correlations of DHA, ARA and LA in mol% in adipose tissue triglycerides (TG), plasma phosphatidylcholine (PC) and erythrocyte PC, respectively, in term infants (TI) and preterm infants without gastrointestinal problems (PTI). Preterm infants with enterostomy (PTI/E) are not shown. All blood and adipose tissue samples were collected during clinically indicated surgery at term-equivalent age. filled diamond = term infants, filled circle = preterm infants

To compare the proportions of DHA, ARA and LA in different compartments, data are demonstrated in Fig. 4. For ARA and DHA, the proportions only correlate between plasma PC and erythrocyte PC, but not between plasma PC and adipose tissue TG as well as between erythrocyte PC and adipose tissue TG. For LA, all proportions correlate well between all three compartments. The concentrations of DHA, ARA and LA in different compartments and groups are summarized in Table 2.

**Table 2:** Concentrations of DHA, ARA and LA in different compartments for all groups

	Adipose tissue TG (µmol/g adipose tissue)			Plasma PC (nmol/ml)			Erythrocytes PC (nmol/ml)		
	DHA	ARA	LA	DHA	ARA	LA	DHA	ARA	LA
TI	12.2 [7.9–17.0]	12.6 [8.7–14.3]	33.3 [24.1–49.4]	95.5 [74.8–124]	275 [235–328]	276 [196–493]	43.2 [35.4–61.3]	176 [132–207]	174 [121–216]
PTI	7.5 [5.9–11.9]	10.2 [8.5–12.1]	14.0 [10.5−17.1]	76 [62.4−102]	236 [220−283]	605 [515–691]	28.9 [23.1–35.8]	105 [93–151]	285 [215–336]
PTI/E	43.4 [32.4–56.9]	10.7 [6.5–12.1]	14.2 [10.5–19.7]	171 [116–175]	205 [197–214]	470 [331–676]	81.8 [43.7–116]	127 [99.9–169]	302 [296–321]

Data are shown as median [25th percentile–75th percentile]. *ARA* arachidonic acid, *DHA* docosahexaenoic acid, *LA* linoleic acid, *PC* phosphatidylcholine, *PTI/E* preterm infants with enterostomy, *PTI* preterm infants without gastrointestinal problems, *TG* triglycerides, *TI* term infants

Data of complete fatty acid analyses of adipose tissue and plasma TG of all patients (online supplement, Table e3) demonstrate substantial differences in the fatty acid composition of these compartments. Particularly, the fraction of ARA (C20:4–N6) in plasma TG was 3.6-fold higher than in adipose tissue TG (*p *< 0.001); whereas there was no significant difference in DHA (C22:6−N3). Moreover, fractions of ARA and DHA were similar in adipose tissue TG (0.88[0.74–1.03]% vs. 0.97[0.67–1.39]%), respectively).

Correlations of postnatal age with the fractions of the major TG fatty acids, palmitic (C16:0), oleic (C18:1−N9) and linoleic (C18:2−N6), and of ARA (C20:4−N6) and DHA (C22:6−N3) are shown in the online supplement, Fig. e1 and e2. While in TG of adipose tissue, the fractions and concentrations of C18:1−N9 and C18:2−N6 increased with increasing postnatal age (Fig. e1A + C), fractions of C16:0, ARA and DHA decrease (Fig. e1A + B). However, absolute concentrations of ARA and DHA remained constant in adipose tissue (Fig. e1D). Such correlations only in part applied to TG of plasma (online supplement Fig. e2).

The proportion of LA increased with postnatal age. This applied for individual PC molecular species, and was strongest for those molecular species containing a palmitic or stearic acid rather than an oleic acid in combination with linoleic acid (see online supplement, Fig. e3A–e6A).

## Discussion

This study investigated the effects of the untimely switch from selective trans-placental fatty acid supply to the fetus to parenteral and enteral nutrition of PTI and PTI/E. We found marked changes in the fatty acid composition of PTIs’ lipid stores, membrane lipids and the main plasma transporters of poly-unsaturated fatty acids ARA/DHA (the phospholipid moiety of lipoproteins) compared to TIs. Our data show that PTIs’ adipose tissue TG at term-equivalent age contain much more LA and less ARA/DHA than that of TI early after birth, whose fatty acid accretion was characterized by preferential placental enrichment of ARA and DHA, and active retention of LA in the mother’s circulation during fetal development [2].

Similar to our findings, Farquahson et al. [25] had previously shown that the content of DHA and ARA in subcutaneous adipose tissue triglycerides decreases in human milk fed term and late preterm infants with increasing postnatal age; whereas linoleic acid increases. Furthermore, feeding formula deficient in DHA resulted in complete depletion of DHA within 10 weeks after birth [25]. Whereas the postnatal decline of DHA and ARA may be physiologic in TI, in PTI this occurs in an untimely fashion (see data and [25, 34]), i.e., before accretion of body lipid stores has even started.

We have demonstrated before that plasma phospholipids of PTI are low in ARA and DHA, but high in LA. This change is completed within 7–10 days after birth, i.e., when full enteral feeding is established [24]. Such alterations in PTI affect plasma phosphatidylethanolamine (PE) as well, which is representative of liver parenchymal stores [24]. Here, we demonstrate that such non-physiological fatty acid pattern, found in plasma phospholipids, uniformly applies to adipose tissue as the major fatty acid reservoir of TI and PTI, and to cell membrane phospholipids, like PC in adipocyte and erythrocyte membranes, as well as to PC in plasma, the predominant transport form of ARA and DHA to supply peripheral organs [20]. Hence, the observed changes in fatty acid composition may be representative for all compartments in PTIs, probably including the brain.

High ARA and DHA content in TG and PC of TI is due to intrauterine enrichment by active placental transport during the last trimester [11]. During this period of development, PTI are cared for in the NICU and fed fortified human milk and formula rich in LA and deficient (for this state of development) in ARA and DHA [25].

In a previous study, we showed that mother’s milk of PTI contained on average 159-mg/l DHA and 300-mg/l ARA, which add up to a daily supply of 24 mg/kg/day of DHA and 45 mg/kg/day of ARA at full feeds (i.e., 150 ml/kg/d mother`s milk). DHA and ARA concentrations seem to be similar in preterm and term infants` breast milk, but the choline concentrations seem to be lower for preterm infant [31]. By contrast, estimates of fetal accretion are 43 mg/kg/day DHA and 140–212 mg/kg/day ARA during the last trimester [31, 35, 36]. Provided that PTI cannot generate DHA and ARA in sufficient quantities, PTI will become DHA and ARA deficient, as standard PTI nutrition only provides 56% and 32% of intrauterine accretion rates.

Of particular concern is that fatty acid composition was remarkably different in the three PTI/E infants (with enterostomy) compared to PTI without gastrointestinal problems. PTI/E infants had much higher proportions of DHA and minimal ARA. These PTI/E received parenteral nutrition for longer periods than PTI without enterostomy. Compared to human milk and formula, parental fat emulsions administered in our institution (Smoflipid^®^ and/or Omegaven^®^) contain more DHA. Either prolonged parental nutrition or the loss of fatty acids via the enterostomy [37] changed the composition of fatty acids in blood and adipose tissue to a non-physiological ARA/DHA ratio.

Notably, supplementation of PTI with DHA alone did not sufficiently adjust fatty acid composition of plasma lipids to adequate, i.e., fetal, values [38]. According to our data, a reduction in LA supply, combined with increased supplementation of DHA and ARA (at a ratio of DHA:ARA of 1:2) in PTI nutrition, may be more appropriate to prevent the observed alterations in lipidome composition and, potentially, to improve outcome of PTI. Importantly, the relevance of ARA supplementation together with that of DHA has previously been identified [39].

Infant formulae have improved over the last years but still differ in their lipid composition from each other and from breast milk and (based on the results presented here) do not meet the requirements of preterm infants. Further research is needed for dose-response, long-term and short-term effects of supplementation on weight and body composition, neurological outcome, inflammation and immunity [40]. Recently the European Union [41] decided that from February 2020 onwards, infant formula has to contain 20–50-mg DHA/100 kcal and does not need to contain ARA. International specialists in infant nutrition published a position paper of the European Academy of Paediatrics and the Child Health Foundation. Based on the available information, they recommended that infant formula should contain ARA in at least equal amounts relative to DHA. But they also pointed out that further well-designed clinical studies should evaluate the optimal intakes of DHA and AA [42, 43]. This meets the opinion of this study group that DHA and ARA should be supplemented in a ratio of 1 to 1–2. This should be combined with the metabolically connected choline [26, 38].

Additionally, the N3RO study showed that exclusive DHA supplementation may even have adverse effects in PTIs, e.g., may be associated with a higher rate of BPD [44].

We suspect that the trans-placental enrichment of ARA/DHA in the fetus, and the active retention of LA in the mother, is important for brain, lung and other organs’ development, and that the inversion of fatty acid profiles observed in this study may contribute to impaired development of PTI.

### Strengths and limitations of the study

In this prospective observational study, the primary goal was to examine the fatty acid composition of adipose tissue, erythrocyte membranes and plasma in preterm infants in comparison to term infants. Unfortunately, only 3 preterm infants with enterostomy could be enrolled (because so few infants required enterostomies during the study period); hence, this group of infants was only analyzed descriptively. Furthermore, the PTI group is dominated by boys due to their higher incidence of inguinal hernia. Finally, it was impossible to measure body composition of the study infants at the time of surgery to calculate pool sizes of individual fatty acids (because these infants required monitoring, nasogastric tubes or respiratory support at that time). But relative molecular composition of fatty acids (in adipose tissue stores and plasma) may be more important for resulting composition of growing organs (like the brain) and better indicators of deterioration of the lipidome than absolute pool size. Strengths of this study are the prospective data and sample collection and handling, and that lipid analyses are based on established methods as demonstrated in previous studies [20, 24].

## Conclusion

(Near-)TI are born with adipose tissue fat depots rich in ARA and DHA. In contrast, adipose tissue of PTI at term-equivalent age contains higher proportions of LA and lower proportions of ARA and DHA. Adipose tissue fatty acid profiles are reflected in PC of plasma and erythrocytes (and probably all other tissues). The observed disturbance in fatty acid composition is likely caused by current PTI nutrition (predominantly human milk and preterm formula, both rich in LA), which is not physiological if compared to the prenatal placental fatty acid accretion. Because of the depot and supply function of adipose tissue for all developing organs including the brain, this non-physiological composition could be harmful for PTI, might contribute to impaired neurocognitive development, and should be addressed in future nutrition concepts.

## Electronic supplementary material

Below is the link to the electronic supplementary material.

Supplementary material 1 (DOC 772 kb)

## Abbreviations

ARA: Arachidonic acid
AT: Adipose tissue
DHA: Docosahexaenoic acid
DTA: Docosatetraenoic acid
ELGANS: Extremely low gestational age newborns
FIP: Focal intestinal perforation
GA: Gestational age
GC: Gas chromatography
LA: Linoleic acid
LC-PUFA: Long-chain poly-unsaturated fatty acid
NEC: Necrotizing enterocolitis
PC: Phosphatidylcholine
PE: Phosphatidylethanolamine
PMA: Postmenstrual age
PNA: Postnatal age
PTI: Preterm infants
PTI/E: Preterm infants with enterostomy
TG: Triglycerides
TI: Term infants
