# Supplementary material for: Fatty acid composition of adipose tissue at term indicates deficiency of arachidonic and docosahexaenoic acid and excessive linoleic acid supply in preterm infants
Source: Eur J Nutr. 2020 May 31;60(2):861–72. doi: 10.1007/s00394-020-02293-2 (PMC7900037; doi:10.1007/s00394-020-02293-2)
Supplement: Supplementary file 1 — Supplementary material 1 (DOC 772 kb) [file 394_2020_2293_MOESM1_ESM.doc]

# Fatty acid composition of adipose tissue at term indicates deficiency of arachidonic and docosahexaenoic acid and excessive linoleic acid supply in preterm infants

# Böckmann K.A.^1^, von Stumpff A.^1^, Bernhard W.^1^, Shunova A.^1^, Minarski M.^1^, Frische B.^1^, Warmann S.^3^, Schleicher E.^4^, Poets C.F.^1^, Franz A.R.^1,2^

# Affiliations: ^1^Department of Neonatology, ^2^Center for Pediatric Clinical Studies, ^3^Department of Pediatric Surgery and Child Urology, ^4^Department of Internal Medicine IV, Faculty of Medicine, Eberhard-Karls-University, Tübingen, Germany.

# Address of correspondence: Katrin Alexandra Böckmann, MD, Department of Neonatology, Faculty of Medicine, Eberhard-Karls-University, Calwer Straße 7, D-72076 Tuebingen, Germany; katrin.boeckmann@med.uni-tuebingen.de; Phone: +49 7071 29 84742

# Short title: fatty acid alterations in preterm infant adipose tissue and blood

# Names for PubMed indexing: Böckmann KA, von Stumpff A, Bernhard W, Shunova A, Minarski M, Frische B, Warmann S, Poets CF, Schleicher E, Franz AR

# Funding: Katrin Böckmann received an internal grant from the Medical Faculty of Tübingen University (AKF 426-0-0).

**Acknowledgements:** The authors wish to thank all children and parents, as well as the nurses and doctors at the Departments of Neonatology and Pediatric Surgery at Tübingen University Children`s Hospital for their support of this project.

| A: Composition | | | | | |
| --- | --- | --- | --- | --- | --- |
| PC Species | Median | 2.5th percentile | 97.5th percentile | Relat. Deviation | |
| PC14:0/22:6 | 0.07 | 0.05 | 0.11 | -0.21 | +0.54 |
| PC16:0/14:0 | 0.33 | 0.22 | 0.58 | -0.33 | +0.76 |
| PC16:0/16:0 | 1.03 | 0.90 | 1.24 | -0.13 | +0.20 |
| PC16:0/16:1 | 1.30 | 1.01 | 1.55 | -0.22 | +0.19 |
| PC16:0/18:0 | 0.52 | 0.42 | 0.67 | -0.20 | +0.29 |
| PC16:0/18:1 | 15.23 | 13.41 | 16.24 | -0.12 | +0.07 |
| PC16:0/18:2 | 27.89 | 25.92 | 28.97 | -0.07 | +0.04 |
| PC16:0/18:3 | 1.42 | 1.18 | 1.54 | -0.17 | +0.08 |
| PC16:0/20:4 | 10.70 | 9.68 | 11.79 | -0.10 | +0.10 |
| PC16:0/20:5 | 1.71 | 1.43 | 2.06 | -0.16 | +0.21 |
| PC16:0/22:6 | 4.00 | 3.44 | 4.55 | -0.14 | +0.14 |
| PC18:0/18:0 | 0.19 | 0.14 | 0.23 | -0.25 | +0.25 |
| PC18:0/18:1 | 2.80 | 2.55 | 3.21 | -0.09 | +0.15 |
| PC18:0/20:4 | 5.64 | 5.18 | 6.53 | -0.08 | +0.16 |
| PC18:0/22:4 | 0.24 | 0.20 | 0.35 | -0.16 | +0.49 |
| PC18:0/22:5 | 0.61 | 0.54 | 0.92 | -0.13 | +0.50 |
| PC18:0/22:6 | 1.30 | 1.10 | 1.79 | -0.15 | +0.37 |
| PC18:0/18:2 | 13.41 | 12.76 | 14.88 | -0.05 | +0.11 |
| PC18:1/18:2 | 8.21 | 7.83 | 8.57 | -0.05 | +0.04 |
| PC18:1/20:4 | 3.36 | 3.21 | 3.90 | -0.05 | +0.16 |
| PC Subgroups |  |  |  |  |  |
| Sat. PC | 2.05 | 1.78 | 2.49 | -0.13 | +0.22 |
| C18:1-PC | 18.11 | 16.20 | 19.33 | -0.11 | +0.07 |
| C18:2-PC | 49.79 | 47.51 | 50.62 | -0.05 | +0.02 |
| C20:4-PC | 19.57 | 18.54 | 21.59 | -0.05 | +0.10 |
| C20:5-PC | 1.71 | 1.43 | 2.06 | -0.16 | +0.21 |
| C22:6-PC | 5.28 | 4.72 | 6.37 | -0.11 | +0.21 |
| Other PC | 3.56 | 2.97 | 4.04 | -0.16 | +0.14 |

| B: Concentration | | | | | |
| --- | --- | --- | --- | --- | --- |
| PC Species | Median | 2.5th percentile | 97.5th percentile | Relat. Deviation | |
| PC14:0/22:6 | 1.23 | 0.98 | 2.02 | -0.20 | +0.64 |
| PC16:0/14:0 | 5.98 | 3.93 | 10.68 | -0.34 | +0.79 |
| PC16:0/16:0 | 18.95 | 15.53 | 21.98 | -0.18 | +0.16 |
| PC16:0/16:1 | 23.77 | 17.95 | 27.31 | -0.24 | +0.15 |
| PC16:0/18:0 | 9.47 | 7.48 | 11.94 | -0.21 | +0.26 |
| PC16:0/18:1 | 276.44 | 242.49 | 288.80 | -0.12 | +0.04 |
| PC16:0/18:2 | 506.10 | 467.13 | 534.10 | -0.08 | +0.06 |
| PC16:0/18:3 | 25.26 | 20.88 | 28.60 | -0.17 | +0.13 |
| PC16:0/20:4 | 193.28 | 170.79 | 215.91 | -0.12 | +0.12 |
| PC16:0/20:5 | 31.29 | 25.39 | 38.30 | -0.19 | +0.22 |
| PC16:0/22:6 | 71.63 | 59.26 | 85.30 | -0.17 | +0.19 |
| PC18:0/18:0 | 3.21 | 2.53 | 4.42 | -0.21 | +0.38 |
| PC18:0/18:1 | 50.36 | 44.98 | 58.56 | -0.11 | +0.16 |
| PC18:0/20:4 | 99.25 | 89.47 | 123.26 | -0.10 | +0.24 |
| PC18:0/22:4 | 4.17 | 3.70 | 6.68 | -0.11 | +0.60 |
| PC18:0/22:5 | 11.25 | 9.37 | 17.46 | -0.17 | +0.55 |
| PC18:0/22:6 | 23.46 | 19.18 | 33.79 | -0.18 | +0.44 |
| PC18:0/18:2 | 248.01 | 222.94 | 270.14 | -0.10 | +0.09 |
| PC18:1/18:2 | 148.94 | 135.89 | 158.70 | -0.09 | +0.07 |
| PC18:1/20:4 | 61.65 | 55.29 | 73.75 | -0.10 | +0.20 |
| PC Subgroups |  |  |  |  |  |
| Sat. PC | 38.27 | 32.07 | 45.26 | -0.16 | +0.18 |
| C18:1-PC | 325.64 | 292.10 | 343.84 | -0.10 | +0.06 |
| C18:2-PC | 894.17 | 846.72 | 939.31 | -0.05 | +0.05 |
| C20:4-PC | 356.57 | 320.43 | 402.28 | -0.10 | +0.13 |
| C20:5-PC | 31.29 | 25.39 | 38.30 | -0.19 | +0.22 |
| C22:6-PC | 94.55 | 80.64 | 120.61 | -0.15 | +0.28 |
| Other PC | 64.38 | 53.23 | 76.44 | -0.17 | +0.19 |
| Total PC | 1797 | 1695 | 1902 | -0.06 | +0.06 |

Table e1, Reproducibility of PC molecular species and sub-group analysis via liquid chromatography-electrospray ionization tandem mass spectrometry (LC-ESI-MS/MS). Data indicate median values, 2.5%/97.5% ranges, and indicate the relative 95% confidence interval of molar composition (A) and concentration (B) of control plasma analyses PC (N=20) during 18 months. Nomenclature indicates phosphatidylcholine (PC) molecular species, where the figures before and after the slash (xy:z/xy:z) indicate the fatty acyl residues in sn-1 or sn-2 position. The figure before the colon (xy) indicates the number of carbon units, and that after the colon(z) the number of double bonds. Subgroups stand for the sum of PC species containing two saturated fatty acyl residues (Sat. PC), or containing an oleic (C18:1-PC), linoleic (C18:2-PC), arachidonic (C20:4-PC) or doscosahexaenoic (C22:6-PC) acid residue. In case of conflict, a molecular species was included in the group containing the more unsaturated fatty acid, like PC18:1/18:2 being included into the C18:2-PC subgroup.

| A: Composition (mol%) | | | | | | | | |
| --- | --- | --- | --- | --- | --- | --- | --- | --- |
| Fatty acid |  | Median | | 2.5th percentile | 97.5th percentile | Relative Deviation | |  |
| Lauric acid | C12:0 | 1.54 | | 1.45 | 1.62 | -0.060 | 0.051 |  |
| Myristic acid | C14:0 | 5.32 | | 5.27 | 5.36 | -0.010 | 0.008 |  |
| Palmitic acid | C16:0 | 32.23 | | 32.09 | 32.41 | -0.004 | 0.006 |  |
| Palmitoleic acid | C16:1-N7 | 8.58 | | 8.48 | 8.67 | -0.011 | 0.011 |  |
| Stearic acid | C18:0 | 5.69 | | 5.63 | 5.78 | -0.011 | 0.015 |  |
| Oleic acid | C18:1-N9 | 22.90 | | 22.80 | 23.03 | -0.005 | 0.006 |  |
| Linoleic acid | C18:2-N6 | 13.79 | | 13.69 | 13.90 | -0.007 | 0.008 |  |
| gamma-Linolenic acid | C18:3-N6 | 0.73 | | 0.63 | 0.93 | -0.138 | 0.275 |  |
| Arachidic acid | C20:0 | 0.15 | | 0.13 | 0.21 | -0.132 | 0.345 |  |
| alpha-Linolenic acid | C18:3-N3 | 0.53 | | 0.48 | 0.60 | -0.098 | 0.132 |  |
| Behenic acid | C22:0 | 0.30 | | 0.26 | 0.37 | -0.143 | 0.223 |  |
| Arachidonic acid | C20:4-N6 | 3.33 | | 3.23 | 3.40 | -0.032 | 0.021 |  |
| Eicosapentaenoic acid | C20:5-N3 | 0.59 | | 0.44 | 0.71 | -0.257 | 0.205 |  |
| Docosatetraenoic acid | C22:4-N6 | 1.29 | | 1.16 | 1.36 | -0.099 | 0.057 |  |
| Docosapentaenoic acid | C22:5-N6 | 0.46 | | 0.43 | 0.52 | -0.072 | 0.126 |  |
| Docosapentaenoic acid | C22:5-N3 | 0.46 | | 0.35 | 0.60 | -0.235 | 0.305 |  |
| Docosahexaenoic acid | C22:6-N3 | 1.45 | | 1.33 | 1.55 | -0.082 | 0.070 |  |
| B: Concentration (mmol/g) | | | | | | | | |
| Fatty acid |  | Median | 2.5th percentile | | 97.5th percentile | Relative Deviation | |  |
| Lauric acid | C12:0 | 9.24 | 8.68 | | 9.69 | -0.061 | 0.049 |  |
| Myristic acid | C14:0 | 31.88 | 31.40 | | 32.40 | -0.015 | 0.016 |  |
| Palmitic acid | C16:0 | 93.13 | 191.47 | | 195.60 | -0.009 | 0.013 |  |
| Palmitoleic acid | C16:1-N7 | 51.54 | 50.58 | | 52.05 | -0.019 | 0.010 |  |
| Stearic acid | C18:0 | 34.17 | 33.69 | | 34.84 | -0.014 | 0.020 |  |
| Oleic acid | C18:1-N9 | 137.48 | 136.05 | | 138.82 | -0.010 | 0.010 |  |
| Linoleic acid | C18:2-N6 | 82.69 | 82.00 | | 83.64 | -0.008 | 0.011 |  |
| gamma-Linolenic acid | C18:3-N6 | 4.42 | 3.80 | | 5.61 | -0.140 | 0.270 |  |
| Arachidic acid | C20:0 | 0.93 | 0.81 | | 1.25 | -0.130 | 0.350 |  |
| alpha-Linolenic acid | C18:3-N3 | 3.22 | 2.90 | | 3.61 | -0.098 | 0.121 |  |
| Behenic acid | C22:0 | 1.81 | 1.55 | | 2.22 | -0.143 | 0.224 |  |
| Arachidonic acid | C20:4-N6 | 20.00 | 19.38 | | 20.52 | -0.031 | 0.026 |  |
| Eicosapentaenoic acid | C20:5-N3 | 3.53 | 2.61 | | 4.25 | -0.261 | 0.204 |  |
| Docosatetraenoic acid | C22:4-N6 | 7.68 | 7.02 | | 8.11 | -0.086 | 0.056 |  |
| Docosapentaenoic acid | C22:5-N6 | 2.79 | 2.58 | | 3.13 | -0.075 | 0.124 |  |
| Docosapentaenoic acid | C22:5-N3 | 2.75 | 2.11 | | 3.59 | -0.235 | 0.305 |  |
| Docosahexaenoic acid | C22:6-N3 | 8.71 | 8.03 | | 9.27 | -0.078 | 0.064 |  |

Table e2, Reproducibility of neutral lipid fatty acid determination via gas chromatography flame ionization detection (GC-FID) after generation of of fatty acid methyl esters (FAME). Data indicate median values, 2.5%/97.5% ranges, and the relative 95% confidence interval of molar composition (A) and concentration (B) of mixted neutral lipid extract of adipose tissue, control plasma and rat liver tissue, using cis-8,11,14-Eicosatrienoic acid (C20:3-N6; mean molecular weight 334.543Da) as internal standard (200µg/mL)

| Fatty acid |  | Adipose tissue | Plasma |
| --- | --- | --- | --- |
| Lauric acid | C12:0 | 1.62 (0.55;3.24) | 1.11 (0.62;2.36) |
| Myristic acid | C14:0 | 5.70 (4.66;7.34) | 2.31 (1.69;3.78) |
| Palmitic acid | C16:0 | 31.7 (27.9;40.0) | 20.7 (19.1;22.2) |
| Palmitoleic acid | C16:1-N7 | 11.5 (9.0;13.7) | 3.87 (2.99;5.76) |
| Stearic acid | C18:0 | 5.19 (4.70;6.66) | 9.77 (7.87;13.23) |
| Oleic acid | C18:1-N9 | 27.9 (27.3;33.4) | 27.2 (25.0;30.8) |
| Linoleic acid | C18:2-N6 | 7.66 (3.02;9.74) | 16.61 (14.14;23.74) |
| γ-Linolenic acid | C18:3-N6 | 0.12 (0.09;0.14) | 0.33 (0.27;0.40) |
| Arachidic acid | C20:0 | 0.12 (0.09;0.13) | 0.21 (0.16;0.27) |
| α-Linolenic acid | C18:3-N3 | 0.51 (0.10;0.86) | 0.94 (0.62;1.26) |
| Eicosatrienoic acid | C20:3-N6 | 0.28 (0.25;0.42) | 0.77 (0.55;1.13) |
| Behenic acid | C22:0 | 0.09 (0.04;0.12) | 0.85 (0.67;1.16) |
| Arachidonic acid | C20:4-N6 | 0.88 (0.74;1.03) | 3.16 (2.52;4.16) |
| Eicosapentaenoic acid | C20:5-N3 | 0.22 (0.13;0.33) | 0.60 (0.42;0.90) |
| Docosatetraenoic acid | C22:4-N6 | 0.23 (0.19;0.30) | 0.32 (0.22;0.59) |
| Docosapentaenoic acid | C22:5-N6 | 0.36 (0.24;0.62) | 0.53 (0.22;1.26) |
| Docosapentaenoic acid | C22:5-N3 | 0.06 (0.04;0.09) | 0.08 (0.06;0.13) |
| Docosahexaenoic acid | C22:6-N3 | 0.97 (0.67;1.39) | 1.26 (0.81;2.23) |
| Ratio C20:4/C22; 6 |  | 0.93 (0.71;1.38) | 3.34 (1.27;4.72) |

Table e3. Fatty acid composition of neutral lipids of adipose tissue and plasma in study patients. Data are median values and interquartile ranges of the neutral lipids of all patients included in the study. Neutral lipids were isolated from organic extracts of adipose tissue and plasma using solid phase NH2-colums, transesterified and analyzed with gas chromatography as described in *Materials and Methods*.

| PC Species | Adipose tissue | Plasma | Erythrocytes |
| --- | --- | --- | --- |
| PC14:0/22:6^###^ | 0.06 (0.04; 0.07) | 0.03 (0.02; 0.03) | 0.03 (0.03; 0.04) |
| PC16:0/14:0* | 2.48 (1.77; 2.85) | 0.37 (0.27; 0.62) | 0.76 (0.60; 0.98) |
| PC16:0/16:0* | 7.09 (6.38; 8.47) | 1.73 (1.51; 1.99) | 6.03 (5.57; 6.63) |
| PC16:0/16:1^†^ | 6.34 (4.77; 9.52) | 1.30 (1.14; 1.73) | 1.81 (1.45; 2.34) |
| PC16:0/18:0* | 1.33 (1.24; 1.54) | 0.79 (0.72; 0.88) | 2.17 (1.96; 2.35) |
| PC16:0/18:1** | 25.20 (21.72; 27.77) | 15.22 (14.31; 19.27) | 25.12 (24.36; 25.94) |
| PC16:0/18:2*** | 12.14 (9.99; 14.81) | 17.25 (13.32; 19.28) | 15.63 (11.96; 17.42) |
| PC16:0/18:3^†^ | 1.00 (0.78; 1.38) | 0.51 (0.41; 0.59) | 0.42 (0.32; 0.54) |
| PC16:0/20:4^#^ | 6.09 (5.43; 7.72) | 9.62 (7.59; 12.93) | 8.21 (6.94; 12.68) |
| PC16:0/20:5^##^ | 0.83 (0.60; 1.00) | 0.68 (0.47; 1.14) | 0.47 (0.36; 0.83) |
| PC16:0/22:6^###^ | 2.00 (1.61; 2.38) | 4.86 (4.05; 6.49) | 3.44 (2.80; 4.62) |
| PC18:0/18:0* | 0.41 (0.37; 0.46) | 0.24 (0.20; 0.27) | 0.32 (0.29; 0.34) |
| PC18:0/18:1** | 7.37 (6.01; 8.51) | 4.57 (3.84; 5.47) | 5.36 (4.99; 5.82) |
| PC18:0/20:4^#^ | 4.89 (4.39; 5.85) | 9.98 (8.12; 12.46) | 7.11 (6.09; 8.98) |
| PC18:0/22:4^†^ | 0.37 (0.29; 0.51) | 0.31 (0.27; 0.40) | 0.30 (0.28; 0.36) |
| PC18:0/22:5^†^ | 0.74 (0.62; 0.84) | 0.91 (0.80; 1.21) | 0.60 (0.51; 0.65) |
| PC18:0/22:6^###^ | 1.11 (0.96; 1.39) | 2.97 (2.50; 4.49) | 1.42 (1.28; 2.03) |
| PC18:0/18:2*** | 7.90 (6.22; 10.40) | 14.06 (9.31; 19.99) | 8.28 (6.21; 12.18) |
| PC18:1/18:2*** | 5.41 (3.96; 6.50) | 8.02 (5.79; 8.85) | 6.66 (5.90; 7.54) |
| PC18:1/20:4^#^ | 2.92 (2.63; 3.45) | 3.03 (2.67; 3.41) | 2.46 (2.30; 2.85) |
| PC Subgroups |  |  |  |
| Sat. PC* | 11.41 (10.24; 12.84) | 3.09 (2.81; 3.57) | 9.51 (8.94; 10.49) |
| C18:1-PC** | 32.97 (27.78; 35.63) | 19.49 (18.23; 23.79) | 30.88 (29.71; 31.78) |
| C18:2-PC*** | 26.56 (20.19; 32.07) | 37.61 (29.52; 45.99) | 30.57 (25.12; 36.12) |
| C20:4-PC^#^ | 14.09 (12.82; 16.15) | 22.78 (18.94; 28.42) | 18.09 (15.41; 23.84) |
| C20:5-PC^##^ | 0.83 (0.60; 1.00) | 0.68 (0.47; 1.14) | 0.47 (0.36; 0.83) |
| C22:6-PC^###^ | 3.25 (2.58; 3.94) | 7.77 (6.54; 10.89) | 4.76 (4.17; 6.87) |
| Other PC^†^ | 0.06 (0.04; 0.07) | 3.16 (2.73; 3.87) | 3.16 (2.86; 3.74) |

Table e4. Molecular species composition of phosphatidylcholine (PC) of adipose tissue, plasma and erythrocytes in study patients Data are median values and interquartile ranges of the analyzed PC molecular species of all patients included in the study. PC was analyzed from organic extracts with liquid chromatography electrospray ionization tandem mass spectrometry LC-ESI-MS/MS) as described in *Materials and Methods*. Symbols (*,**,***,^#^,^##^,^###^,^†^) indicate which molecular species served for the generation of the respective PC subgroups.


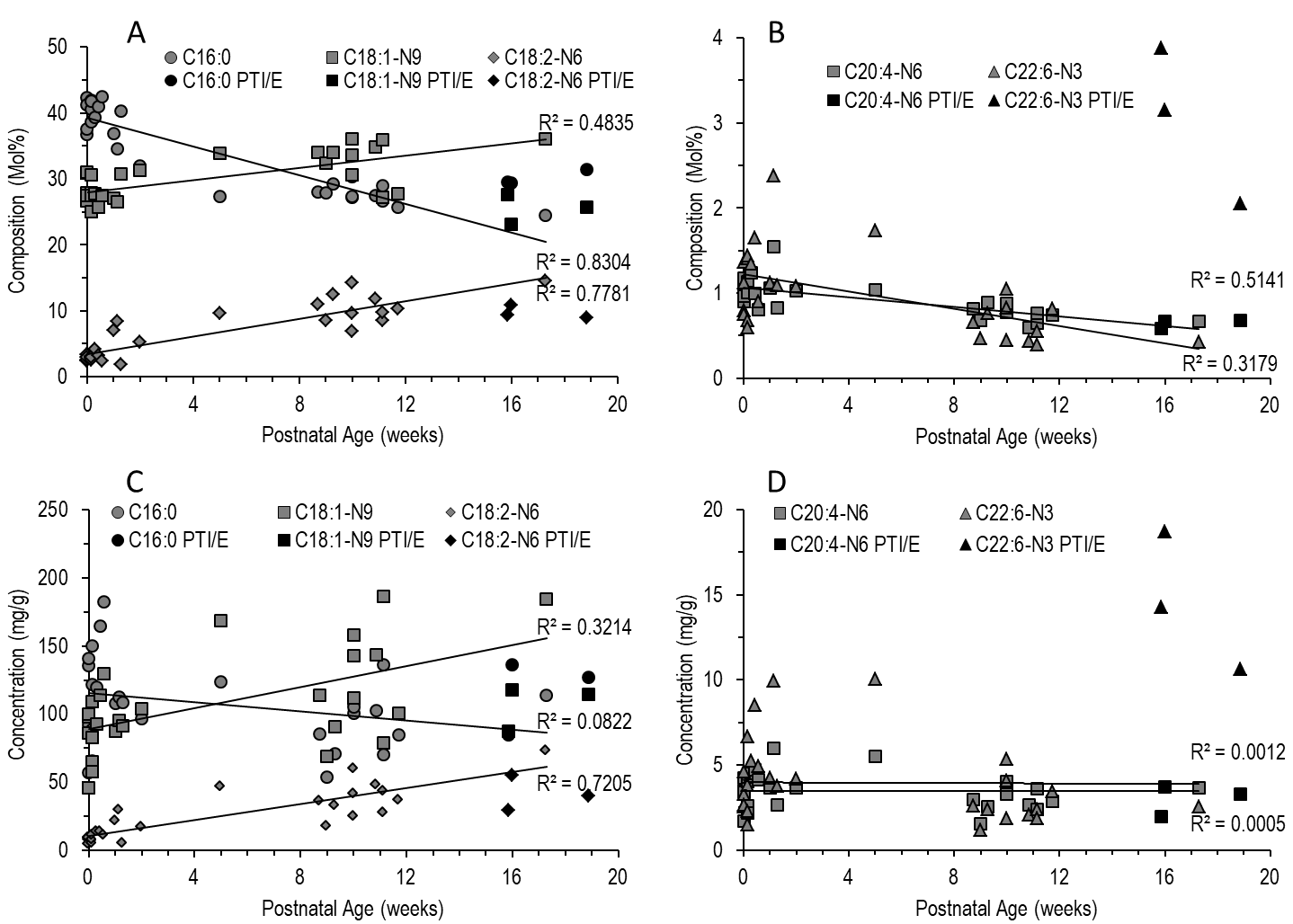


Figure e1. Fatty acid composition of adipose tissue triglycerides. plasma and erythrocytes Data are values of fatty acid fractions (A, B) or concentrations (C, D) of all patients relative to postnatal age. Regression curves were calculated from term (TI, 0-2wk) and preterm (PTI, >4wk postnatal age) infants. Data of preterm infants with enterostoma receiving long term parenteral nutrition (PTI/E) are separately indicated. Fatty acids were analyzed with gas chromatorgraphy as described in *Materials and Methods*. Abbreviations: C16:0, palmitic acid; C18:1N-9, oleic acid; C18:2N-6, linoleic acid; C20:4N-6, arachidonic acid; C22; 6N-3, docosahexaenoic acid.


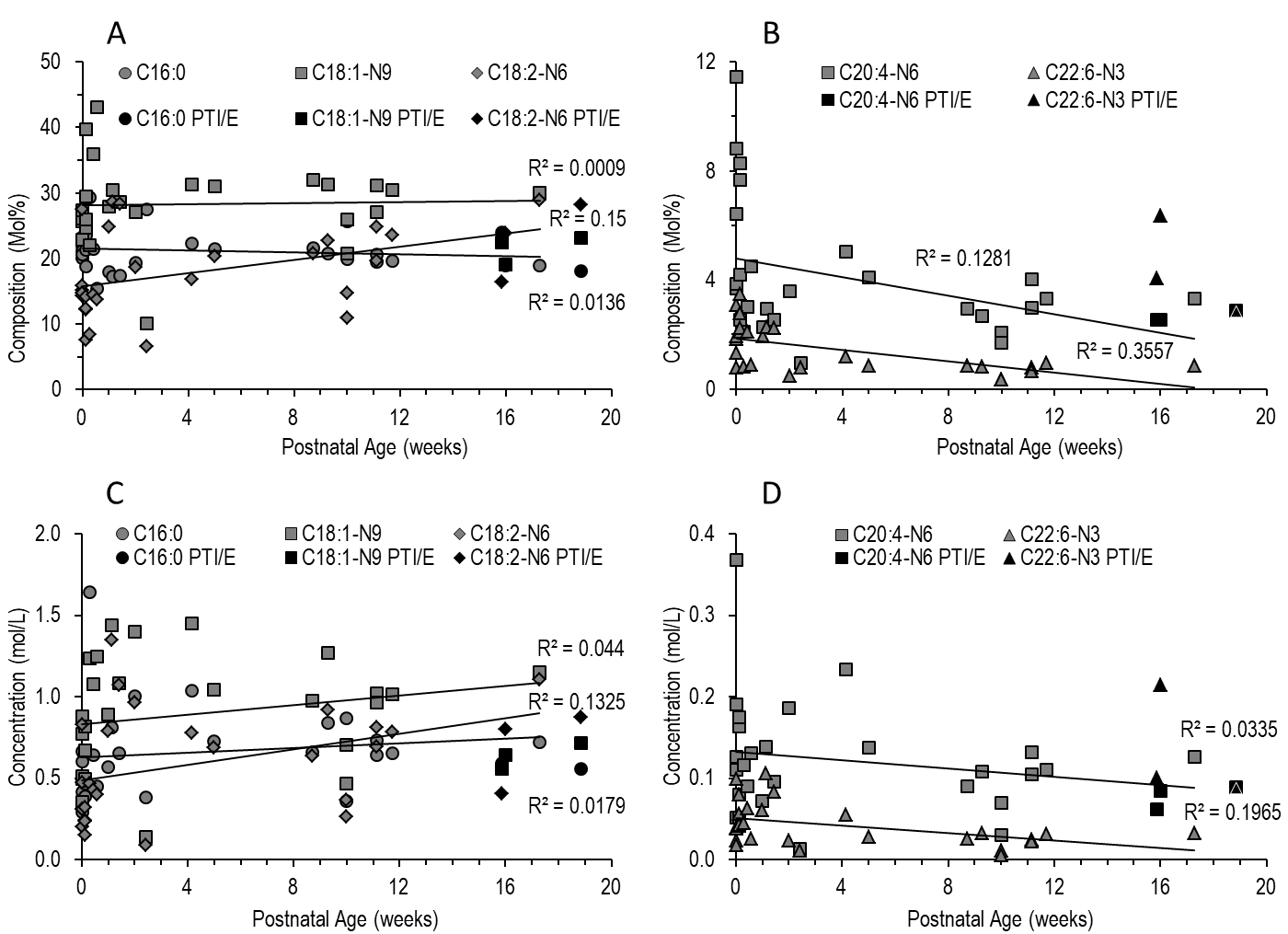


Figure e2. Fatty acid composition of plasma triglycerides tissue neutral lipids. plasma and erythrocytes Data are values of fatty acid fractions (A, B) or concentrations (C, D) of all patients relative to postnatal age. Regression curves were calculated from term (TI, 0-2wk) and preterm (PTI, >4wk postnatal age) infants. Data of preterm infants with enterostoma receiving long term parenteral nutrition (PTI/E) are separately indicated. Fatty acids were analyzed with gas chromatorgraphy as described in *Materials and Methods*. Abbreviations: C16:0, palmitic acid; C18:1N-9, oleic acid; C18:2N-6, linoleic acid; C20:4N-6, arachidonic acid; C22; 6N-3, docosahexaenoic acid.


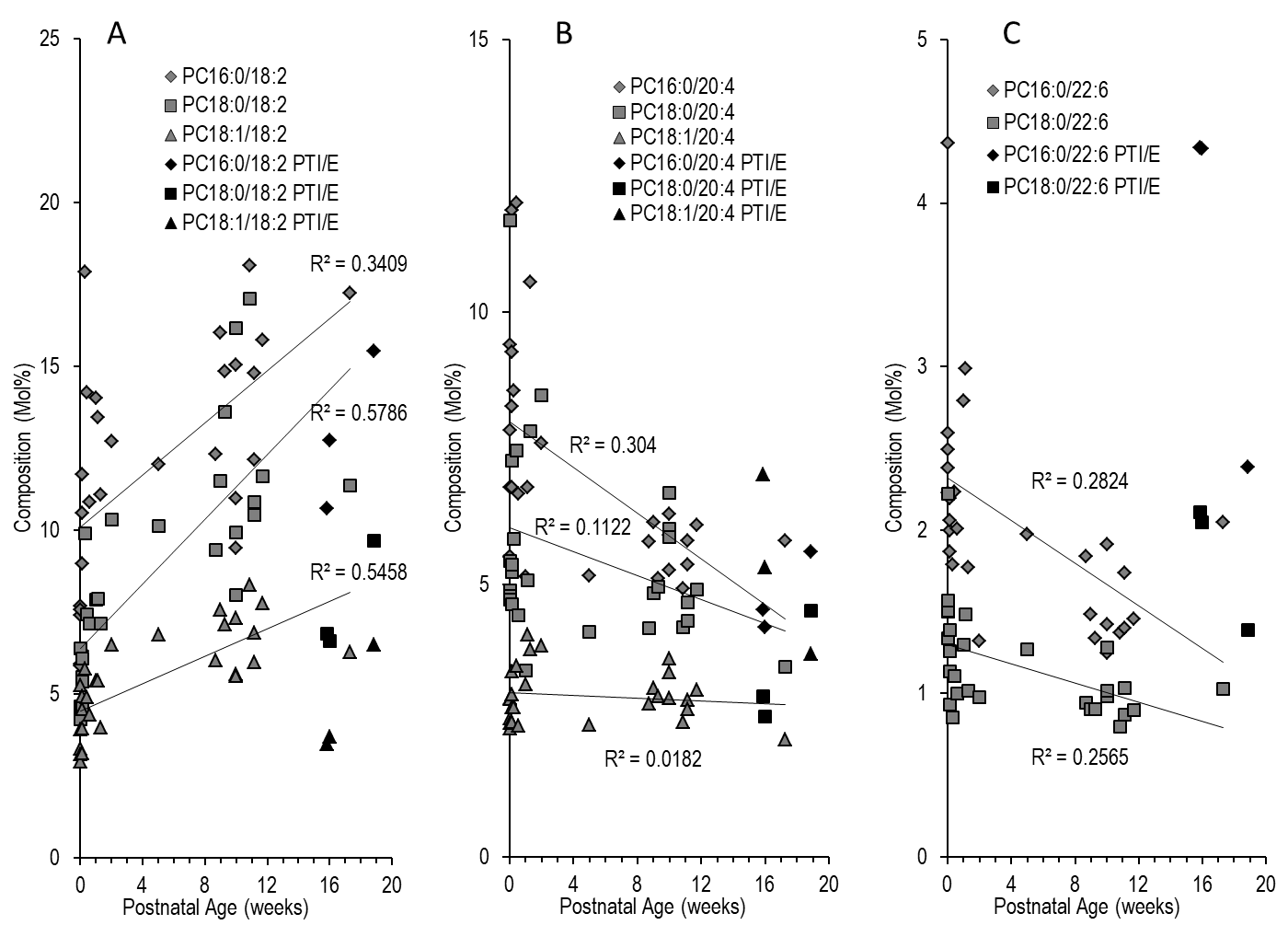


Figure e3. Postnatal changes of PC molecular species composition in adipose tissue. PC was analyzed from organic extracts with liquid chromatography electrospray ionization tandem mass spectrometry LC-ESI-MS/MS) as described in *Materials and Methods*. Regression curves are calculated from term (TI, 0-2wk) and preterm (PTI, >4wk postnatal age) infants. Data of preterm infants with enterostoma receiving long term parenteral nutrition (PTI/E) are separately indicated. Abbreviations: PC16:0/18:2, palmitoyl-linoleoyl-PC; PC18:0/18:2, stearoyl-linoleoyl-PC; PC18:1/18:2, oleoyl-linoleoyl-PC; PC16:0/20:4, palmitoyl-arachidonoyl-PC; PC18:0/20:4, stearoyl-arachidonoyl-PC; PC18:1/20:4, oleoyl-arachidonoyl-PC; PC16:0/22:6 , palmitoyl-docosaheaenoyxl-PC; PC18:0/22:6, stearoyl-docosaheaenoyxl-PC; R, coefficient of determination.


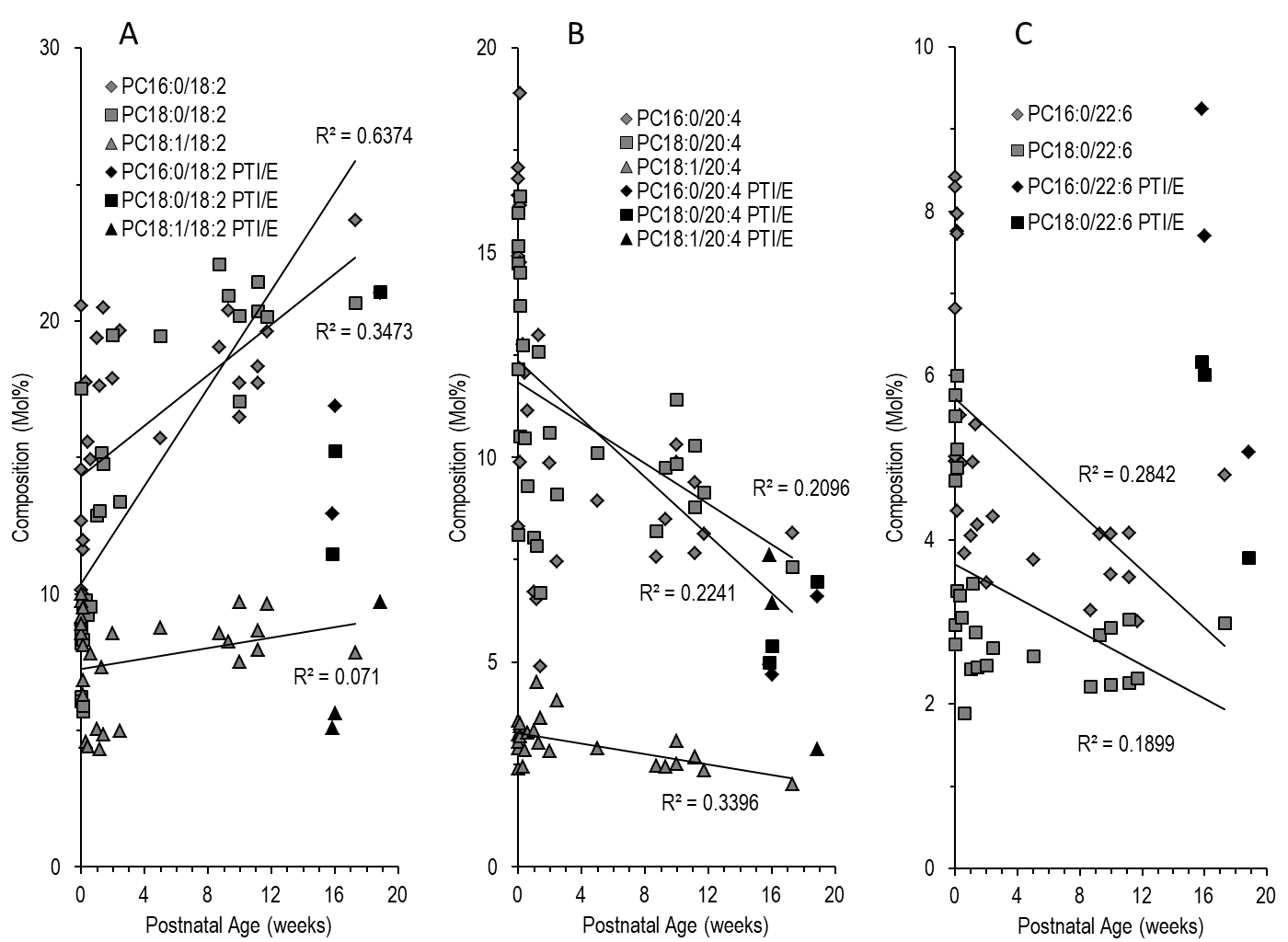


Figure e4. Postnatal changes of PC molecular species composition in plasma. PC was analyzed from organic extracts with liquid chromatography electrospray ionization tandem mass spectrometry LC-ESI-MS/MS) as described in *Materials and Methods*. Regression curves are calculated from term (TI, 0-2wk) and preterm (PTI, >4wk postnatal age) infants. Data of preterm infants with enterostoma receiving long term parenteral nutrition (PTI/E) are separately indicated. Abbreviations: see legend to Figure e3


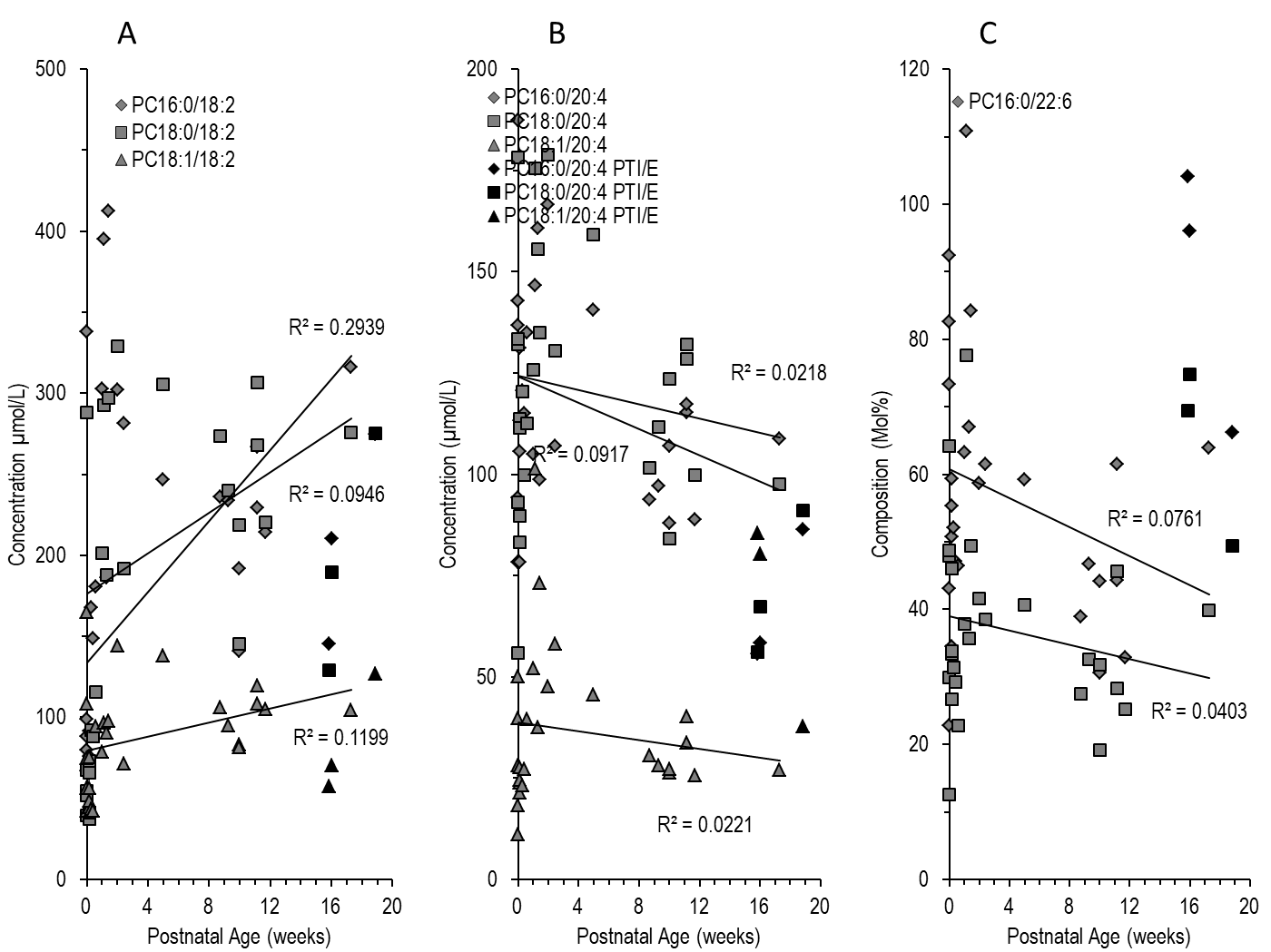


Figure e5. Postnatal changes of PC molecular species concentrations in plasma. PC was analyzed from organic extracts with liquid chromatography electrospray ionization tandem mass spectrometry LC-ESI-MS/MS) as described in *Materials and Methods*. Regression curves are calculated from term (TI, 0-2wk) and preterm (PTI, >4wk postnatal age) infants. Data of preterm infants with enterostoma receiving long term parenteral nutrition (PTI/E) are separately indicated. Abbreviations: see legend to Figure e3.


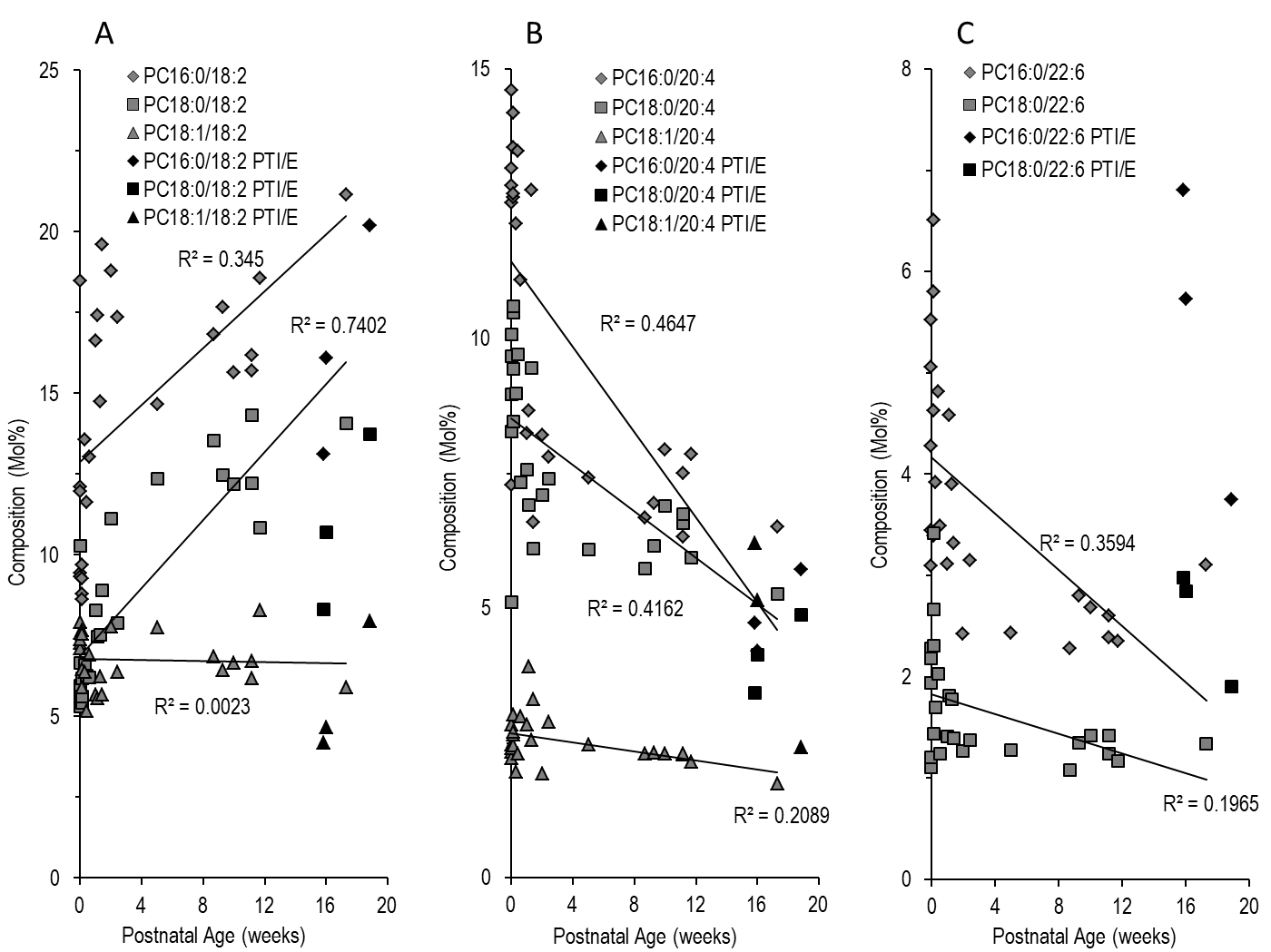


Figure e6. Postnatal changes of PC molecular species concentrations in erythrocyte membranes. PC was analyzed from organic extracts with liquid chromatography electrospray ionization tandem mass spectrometry LC-ESI-MS/MS) as described in Materials and Methods. Regression curves are calculated from term (TI, 0-2wk) and preterm (PTI, >4wk postnatal age) infants. Data of preterm infants with enterostoma receiving long term parenteral nutrition (PTI/E) are separately indicated. Abbreviations: see legend to Figure e3.
